# Integrin β4 promotes cell invasion and epithelial-mesenchymal transition through the modulation of Slug expression in hepatocellular carcinoma

**DOI:** 10.1038/srep40464

**Published:** 2017-01-13

**Authors:** Xiao-Long Li, Lin Liu, Dan-Dan Li, Ya-Ping He, Le-Hang Guo, Li-Ping Sun, Lin-Na Liu, Hui-Xiong Xu, Xiao-Ping Zhang

**Affiliations:** 1 Department of Medical Ultrasound, Shanghai Tenth People’s Hospital, Ultrasound Research and Educational Institute, Tongji University School of Medicine, Shanghai 200072, China; 2 Department of Interventional & Vascular Surgery, Tongji University School of Medicine, Shanghai 200072, China

## Abstract

Integrin β4 (ITGB4) is a transmembrane receptor involved in tumorigenesis and the invasiveness of many cancers. However, its role in hepatocellular carcinoma (HCC), one of the most prevalent human cancers worldwide, remains unclear. Here, we examined the involvement of ITGB4 in HCC and explored the underlying mechanisms. Real-time PCR and immunohistochemical analyses of tissues from 82 patients with HCC and four HCC cell lines showed higher ITGB4 levels in tumor than in adjacent non-tumor tissues and in HCC than in normal hepatic cells. Silencing of ITGB4 repressed cell proliferation, colony forming ability and cell invasiveness, whereas ectopic expression of ITGB4 promoted the proliferation and invasion of HCC cells and induced epithelial to mesenchymal transition (EMT) in parallel with the upregulation of Slug, as shown by transwell assays, WB and immunocytochemistry. Knockdown of Slug reduced cell viability inhibited invasion and reversed the effects of ITBG4 overexpression on promoting EMT, and AKT/Sox2-Nanog may also be involved. In a xenograft tumor model induced by injection of ITGB4-overexpressing cells into nude mice, ITGB4 promoted tumor growth and metastasis to the lungs. Taken together, our results indicate that ITGB4 plays a tumorigenic and pro-metastatic role mediated by Slug and suggest IGTB4 could be a prognostic indicator or a therapeutic target in patients with HCC.

Hepatocellular carcinoma (HCC) is one of the most prevalent human cancers and the third leading cause of cancer-related deaths worldwide^1^. HCC is mainly attributed to viral hepatitis infection and metabolic toxins such as alcohol or aflatoxin, but it can also be caused by conditions like hemochromatosis, α1-antitrypsin deficiency and non-alcoholic steatohepatitis^2^,^3^. The pathogenesis of HCC is a multistep process that involves many genetic or epigenetic alterations, leading to the malignant transformation of hepatocytes^4^. Despite significant advances in the diagnosis and management of HCC, the 5-year overall survival of HCC patients remains poor, with metastasis as the main reason for the high mortality rates after surgery^5^. The mechanisms underlying the development and progression of HCC are not fully understood, underscoring the need to identify molecular markers and therapeutic targets for the treatment of patients with HCC.

Integrins are a large family of heterodimeric transmembrane receptors that mediate cell attachment to other cells or to the extracellular matrix via interactions with proteins such as fibronectin and collagen. Integrins are heterodimers composed of an α and a β subunit, and they play important roles in many physiological and pathological processes, including cell adhesion, migration, proliferation, differentiation, and tumor progression^6^. Integrin β4 (ITGB4) is a laminin-5 receptor that is predominantly expressed in squamous epithelial cells, endothelial cells, immature thymocytes, Schwann cells, and fibroblasts of the peripheral nervous system^7^. The ITGB4 subunit, which is characterized by its particularly long cytoplasmic signaling domain, pairs only with the α6 subunit, and the heterodimeric integrin α6β4 plays a role in the invasive and metastatic phenotype of various cancers^8^,^9^. This tumorigenic role of integrin α6β4 is mediated by the phosphorylation of the cytoplasmic tail of ITGB4, which releases integrin α6β4 from hemidesmosomes, leading to its interaction with growth factor receptors and the induction of growth signaling^10^,^11^. Integrin α6β4 binding to laminin activates phosphoinositide-3-kinase (PI3K) and RhoA small GTPases. In addition, integrin α6β4 interacts with growth factor receptors including those of the epidermal growth factor receptor family to activate signaling pathways involved in tumorigenesis and metastasis, including PI3K, AKT, and MAPK signaling. In addition, ITGB4 is upregulated and associated with tumor invasiveness in squamous cell carcinomas and papillary carcinomas of the thyroid, and it is associated with poor prognosis in breast and bladder cancers^12^,^13^,^14^,^15^. In tumor tissues, the phosphorylation of the cytoplasmic tail of ITGB4 leads to its release from hemidesmosomes and its interaction with growth factor receptors, which promotes the invasion and metastasis of tumor cells^11^.

Epithelial to mesenchymal transition (EMT) is the process by which cells lose their epithelial phenotype and acquire the characteristics of mesenchymal cells^16^. During the process of EMT, cells lose their adhesive properties and undergo alterations in polarity and reorganization of the cytoskeleton in association with the upregulation of extracellular matrix components and the acquisition of migratory and invasive properties^17^. The process of EMT is modulated by transcription factors such as Snail, Slug (Snai2), Twist, Zeb and Foxc2, which have been associated with tumor invasion and metastasis^18^. Pluripotency is the ability of a cell to differentiate into any cell type and is a unique characteristic of embryonic stem cells (ESCs)^19^. Pluripotency transcription factors such as Sox2, Nanog, KLF4 and c-MYC, etc have been also suggested to be oncogenes and may be implicated in the development of several cancers involving multiple signaling pathways including PI3K, AKT, etc^20^,^21^,^22^,^23^,^24^. Previous reports have shown that the aforementioned transcription factors are regulated, at least in part, by pluripotency factors^19^,^25^. As one of these EMT-inducing transcription factors, Slug is upregulated in numerous cancers including lung cancer, hepatocellular carcinoma, leukemia etc^26^. And it also has been shown to associate with a broad spectrum of biological functions in tumor cells such as cell invasion, metastasis, which can activate signaling networks that facilitate the disruption of cell-cell adherens junctions and cell-ECM adhesions mediated by integrins^27^,^28^. As reprogramming-related genes, Sox2 and Nanog were reported to promote HCC progression with the stimulation of Slug^29^, which modulate EMT and metastasis by binding to Slug promoter and transcriptionally regulate Slug expression in tumor cells^30^,^31^. However, whether ITGB4 has a role in this mechanism was unclear.

As ITGB4 is associated with promoting the invasion and metastasis of tumor cells in several cancers, in the present study, we examined the role if ITGB4 in HCC *in vitro* and *in vivo* and explored the underlying mechanisms. Our results indicated that ITGB4 induces HCC cell growth and invasiveness and promotes EMT via a mechanism involving the transcription factor Slug. ITGB4 promoted tumor growth in a xenograft tumor model *in vivo*. Taken together, our results reveal a novel regulatory mechanism for ITGB4 expression and suggest potential therapeutic targets for patients with HCC.

## Materials and Methods

### Patients and Tissues

Biopsies of HCC tissues were collected from 82 patients receiving curative resection at the Shanghai 10th People’s Hospital, affiliated to Tongji University, Shanghai, between 2012 and 2015. Normal adjacent tissues were collected as controls. None of the patients received preoperative therapy and informed consent was obtained from all patients. This study was conducted in accordance with the Declaration of Helsinki and approved by the Ethics Committee of Tongji University. Clinicopathological parameters of study subjects are shown in Table 1.

### Cell culture

Four human HCC cell lines including two highly metastatic potential cell lines (MHCC-97H, MHCC-LM3) and two common epithelial-like cell lines (Bel-7402, SMMC-7721), one normal human hepatic cell line (L02) and HEK293T cells were all purchased from the Type Culture Collection of the Chinese Academy of Sciences (Shanghai, China). All cell lines were cultured in Dulbecco’s Modified Eagles Medium (DMEM) (Gibco, Carlsbad, CA, USA) supplemented with 10% fetal bovine serum (FBS) (Life Technology, Grand Island, NY, USA), 100 IU/mL of penicillin and 100 μg/mL of streptomycin at 37 °C in 5% CO_2_.

### RNA extraction, reverse transcription, and real-time RT-PCR

Total RNA was extracted from tissues and cells using TRIzol reagent (Invitrogen, Carlsbad, CA, USA) according to the manufacturer’s protocol. ITGB4 mRNA expression was measured by regular and real-time reverse transcription (RT) PCR. 1 μg of RNA was reversed transcribed using an oligo-dT primer (Takara) and reverse transcriptase. SYBR green detection was used for real-time PCR with the Stratagene Mx3000 P Real-Time PCR system (Agilent Technologies, Santa Clara, CA). Reactions were incubated at 95 °C for 10 min followed by 40 cycles of 95 °C for 10 s, 55 °C for 30 s and 72 °C for 1 min. The 2−ΔΔCt method was used to measure the fold changes of mRNA expression. The following primer sequences were used: ITGB4 forward primer, GCTTCACACCTATTTCCCTGTC; ITGB4 reverse primer, GACCCAGTCCTCGTCTTCTG; GAPDH forward primer, CTGGGCTACACTGAGCACC; GAPDH reverse primer, AAGTGGTCGTTGAGGGCAATG.

### Immunohistochemistry

Formalin-fixed tumor tissue was embedded in paraffin and sectioned using a microtome. Tissue sections were de-paraffinized using xylene and rehydrated through an ethanol series. Antigen retrieval was performed by boiling in citrate buffer (pH 6) for 15 min. Endogenous peroxidase activity was inhibited by incubating in 3% H_2_O_2_ for 5 min at room temperature. After washing in PBS, non-specific binding sites were blocked using goat serum for 20 min at room temperature before incubating in anti-ITGB4 antibody (Proteintech Group Inc, Chicago, IL, USA) and anti-SNAI2 polyclonal antibody (Proteintech Group Inc, Chicago, IL, USA) overnight at 4 °C. After washing in PBS, sections were labeled with biotinylated secondary antibodies for 30 min at room temperature and immune complexes were amplified using a streptavidin-peroxidase complex and detected using 3, 3′-diaminobenzine solution (DAKO). Tissue sections were counterstained with hematoxylin, dehydrated and mounted in Malinol (Muto Pure Chemicals).

### Stable overexpression of ITGB4 and ITGB4 shRNA in cell lines

The PCR products of ITGB4 were sub-cloned into the lentiviral expression vector pCDH-CMV-EF1-copGFP (SBI, Mountain View, CA, USA) following the manufacturer protocol. And ITGB4 was stably expressed in cell lines. To knock down ITGB4, three short hairpin RNA (shRNA) sequences (sh-1#: 5′-CTTAAAGCCCACCATGTGACC-3′; sh-2#: 5-TTCTCTGTAAGCTTCAGCAGG-3′; sh-3#: 5′-TGGCTGTGATGACAACATCT-3′) were designed and synthesized by GenePharma (Shanghai, China). A scramble negative shRNA was used as a control (SCR: 5′-GACCTGTACGCCAACACAGTG-3′). The sequences were cloned using the lentiviral pLKO.1 puro vector. The Lentiviral vectors were purchased from Zhongqing Co., Ltd (Suzhou, Jiangsu, China) and lentiviral production and transduction were performed according to the manufacturer’s protocol.

### Knockdown of Slug expression

Double-stranded small interfering RNA (siRNA) molecules targeting Slug were purchased from Dharmacon (Dharmacon, Lafayette, CO, USA) (si-Slug: 5′-TGTCCTTGAAGCAACCAGGGT-3′). The media were changed after cells were transfected with 10 nmol/L scrambled siRNA (SCR) or Slug siRNA for 6 h using Lipofectamine TM 2000 reagent (Invitrogen, Carlsbad, CA, USA) at 37 °C following the manufacturer’s instructions. And then the transfection efficiency was detected after 72 h.

### MTT and colony formation assays

Cell proliferation was measured in ITGB4-overexpressing cells using the Cell Proliferation Reagent Kit I (MTT) (Roche, Basel, Switzerland). Stably transfected cells were seeded into 96-well plates (4 × 10^3^/well) in maintenance medium, and cell proliferation was monitored every 24 h, according to the manufacturer’s instructions. For the colony formation assay, stably transfected cells were seeded into six-well plates (500 cells/well) and maintained in DMEM plus 12% FBS for 12 days, with media changes every 4 days. Colonies were fixed with methanol and stained with 0.1% crystal violet (Sigma-Aldrich, St. Louis, MO, USA) in PBS for 15 min. The number of stained colonies per well were quantified in triplicate for each treatment group.

### Cell invasion assays

To measure the effect of ITGB4 on cell migration and invasion, 5 × 10^4^ stably transfected HCC cells were seeded into the upper chamber of an insert coated with Matrigel (Sigma-Aldrich). Cells in the upper chamber were cultured in serum-free media, while medium containing 10% FBS was placed in the lower chamber. The upper and lower chambers were separated by polycarbonate membranes with a pore size of 8 μm (Corning Inc, NY). After incubating for 24 h, cells on the upper membrane were removed with cotton wool and cells that had migrated through the membrane to invade the lower chamber were fixed with methanol and stained in 0.1% cresyl violet. The stained cells were visualized using an IX71 inverted microscope (Olympus, Tokyo, Japan) and the number of stained cells was quantified.

### Western blotting

For western blotting, stably transfected cells were lysed in 50 mM Tris-HCl (pH 7.4), 150 mM NaCl, 1 mM EDTA and 1% Triton X-100 with protease/phosphatase inhibitors (Sigma-Aldrich). Cell lysates were separated by SDS-PAGE and transferred onto a PVDF membrane. Membranes were labeled with primary antibodies against E-cadherin, N-cadherin, Vimentin, AKT, Sox2, Nanog and GAPDH (Cell Signaling Technology, Beverly, MA, USA), followed by horseradish peroxidase (HRP)-conjugated secondary antibodies (Cell Signaling Technology). Protein bands were visualized using a peroxidase substrate for enhanced chemoluminescence (Thermo Scientific).

### Immunofluorescence

For immunofluorescence staining, cells were washed with PBS and fixed in 2% formalin. The cells were permeabilized with PBS/0.5% TX-100 for 10 min at 4 °C and washed three times with 100 mM glycine in PBS for 10–15 min at room temperature. Non-specific antigen binding sites were blocked in 10% goat serum in a PBS solution containing 7.7 mM sodium azide, 0.1% bovine serum albumin, 0.2% TX-100 and 0.05% Tween-20 for 1–2 h at room temperature. After blocking, the cells were incubated in primary antibodies diluted in blocking buffer overnight at 4 °C. Cells were washed three times for 20 min each in the PBS/sodium azide solution prior to labeling with Alexa-fluorescent-conjugated secondary antibodies (Molecular Probes), diluted 1:200 in blocking buffer, for 45–60 min at room temperature. Cells were then washed three times for 20 min each in PBS solution. Nuclei were stained using TOPRO-3 (5 μM) and DAPI (0.5 ng/mL) for 15–30 min. Cells were mounted for imaging with Prolong Antifade Gold and staining was visualized on a Zeiss LSM 510 confocal microscope.

### *In vivo* tumor growth model

Nude mice (4–6 weeks of age, 18–20 g) were purchased from the Shanghai Slaccas Animal Center (Shanghai, China). All animal studies were approved by the Ethics Committee of Shanghai 10th People’s Hospital. All mice were housed in specific pathogen-free conditions following the guidelines of the Institutional Animal Care and Use Committee of Tongji University School of Medicine. The mice were randomly divided into at least six mice in each group. Bel-7402 cells were transduced with empty vectors or vectors expressing ITGB4 in 150 μl of serum-free medium and injected subcutaneously (5 × 10^6^ cells/mouse). Tumor formation was measured once a week using calipers and the tumor volume (V) was calculated using the following formula: (width × 2) × (length)/2.

To analyze the metastasis of tumors, we used the tail vein metastasis model. Bel-7402 cells (5 × 10^5^ cells/mouse) infected with empty vectors or vectors expressing ITGB4 in 100 μl of sterile PBS were injected into the tail vein of six-week old nude mice. The animals were maintained for five weeks in a sterile animal facility. The mice were then sacrificed and the tumors were excised, weighed and measured.

### Statistical analysis

Data are presented as the mean ± standard deviation (SD) of three independent experiments and statistical analyses were performed using SPSS 16.0 for Windows. The two-tailed Student’s t-test was used to analyze statistical differences between groups. P values less than 0.05 were considered statistically significant.

## Results

### ITGB4 expression is upregulated in HCC tissues and HCC cell lines

**Figure 1:**
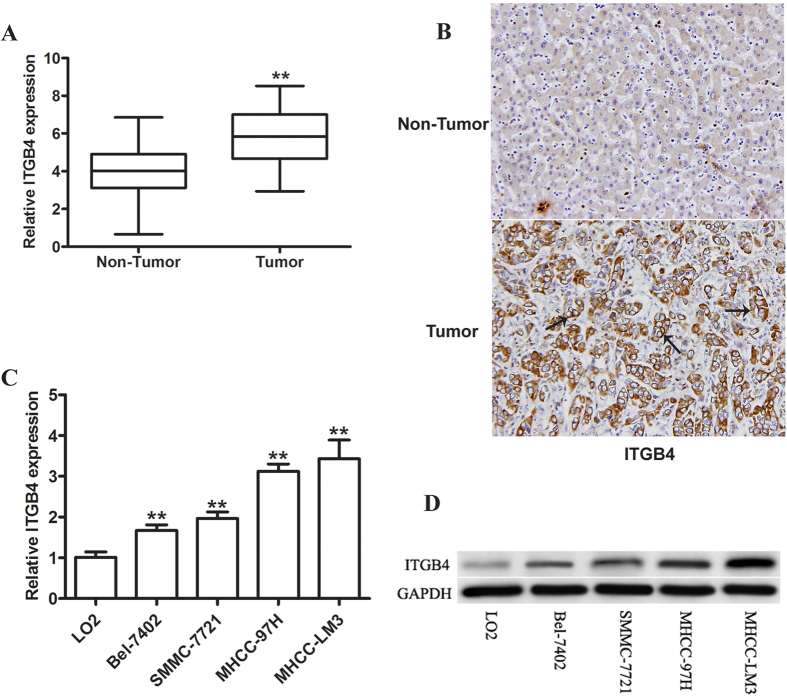
ITGB4 is overexpressed in HCC tissues and cell lines. (**A**) Real-time qPCR analysis of ITGB4 expression in paired HCC tumor and matched normal adjacent tissue samples. n = 82, **p < 0.01. (**B**) Immunohistochemical staining for ITGB4 in HCC primary tumors and matched normal adjacent tissue samples revealed greater amounts of ITGB4 in HCC tissue (original magnification: 200 × ). (**C,D**) Real-time PCR and western blot assessment of ITGB4 mRNA and protein expression in HCC cell lines (Bel-7402, SMMC-7721, MHCC-97H, and MHCC-LM3) and normal hepatic cells (LO2), and MHCC-LM3 and MHCC-97H lines showed the highest levels of ITGB4 expression. GAPDH was used as a loading control. n = 3, **p < 0.01.

The expression of ITGB4 was assessed in paired tumor and adjacent non-tumor samples from 82 patients with HCC by immunohistochemical staining and real-time qPCR. The qRT-PCR results showed significantly higher expression of ITGB4 in tumor than in adjacent non-tumor tissues (P < 0.01, Fig. 1A). Consistent with these results, immunohistochemical staining showed that, in 48 out of the 82 tissues pairs, a higher expression of ITGB4 was found in tumor than in non-tumor tissues (Fig. 1B). Moreover, as Table 1 showed, the high expression of ITGB4 in HCC tumor issues was significantly correlated with stage (P = 0.024) and vascular invasion (P = 0.016). RT-PCR and western blot analyses of ITGB4 expression in the HCC cell lines Bel-7402, SMMC-7721, MHCC-97H, and MHCC-LM3 in comparison to the LO2 normal hepatic cell line showed significantly higher ITGB4 expression in HCC than in normal cells at the mRNA (Fig. 1C) and protein (Fig. 1D) levels. The MHCC-LM3 and MHCC-97H lines showed the highest levels of ITGB4 expression and were therefore used for silencing experiments.

### Silencing of ITGB4 inhibits HCC cell proliferation, invasion, and colony formation

**Figure 2:**
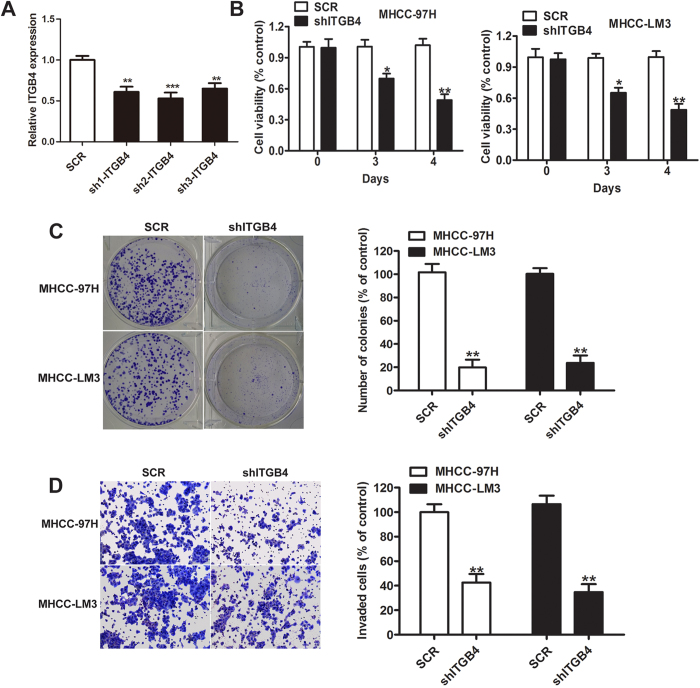
Effects of ITGB4 silencing on HCC cell proliferation, invasion, and colony formation. (**A**) The knockdown efficiency of ITGB4 by qRT-PCR analysis, the ratios of sh1-ITGB4, sh2-ITGB4 and sh3-ITGB4 were approximately 0.61, 0.46 and 0.58 compared with scrambled siRNA (SCR). **p < 0.01, ***p < 0.001. (**B**) ITGB4 shRNA was stably expressed in the MHCC-97H and MHCC-LM3 cell lines, and cell viability was assessed using the MTT assay, and showed significantly lower rates of cell proliferation compared with negative control (NC). (**C**) Colony forming ability decreased in cells treated with shITGB4, which was determined by crystal violet staining and quantified by counting the number of colonies. (**D**) Compared with negative control, invasive ability of shITGB4-transduced cells was also decreased determined by the Transwell assay and quantified after 24 h, respectively. *P < 0.05, **P < 0.01.

To examine the role of ITGB4 in HCC, MHCC-LM3 and MHCC-97H cells were infected with a lentiviral vector expressing shRNA against ITGB4 to determine the effects of ITGB4 knockdown as Fig. 2A showed. Silencing of ITGB4 significantly inhibited HCC cell proliferation, as determined by the MTT assay, in both cell lines (Fig. 2B, P < 0.05 at 3 days and P < 0.01 at 4 days). Colony formation assays showed that ITGB4 knockdown significantly inhibited colony forming ability by approximately 80% in both cell lines (Fig. 2C). Transwell invasion assays showed an approximately 55–60% inhibition of cell invasion in shITGB4 expressing cells compared with controls (Fig. 2D, P < 0.01). Taken together, these results indicate that ITGB4 promotes HCC cell proliferation, colony formation and invasion.

### ITGB4 promotes the proliferation, invasion and colony formation of HCC cells and induces epithelial-mesenchymal transition by modulating Slug expression

**Figure 3:**
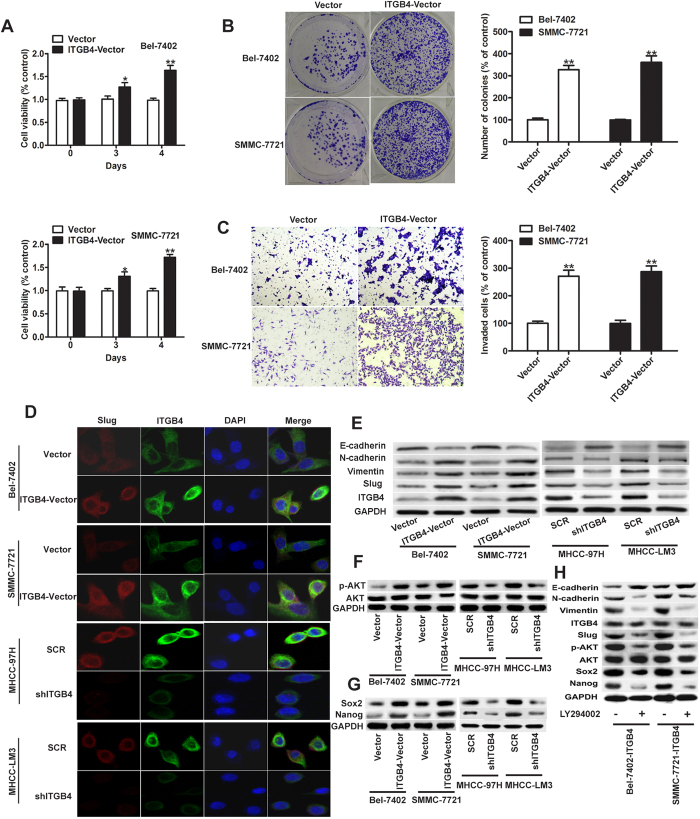
Effects of ITGB4 overexpression on HCC cell proliferation, colony formation, invasion and EMT. (**A**) ITGB4 was ectopically expressed in the Bel-7402 and SMMC-7721 cell lines, and cell viability was assessed using the MTT assay, and showed significantly higher rates of cell proliferation compared with negative control. (**B**) Colony forming ability increased in cells treated with ITGB4 vector, which was determined by crystal violet staining and quantified by counting the number of colonies. (**C**) Compared with negative control, invasive ability of ITGB4-transduced cells was increased determined by the Transwell assay and quantified after 24 h, respectively. (**D**) Representative images of immunofluorescent detection of ITGB4 and Slug expression in ITGB4-overexpressing cells (Bel-7402 and SMMC-7721), sh-ITGB4 cells (MHCC-97H and MHCC-LM3) and matched control cells. Nuclei were counterstained using DAPI. The results demonstrated an increasing expression of ITGB4 and Slug mainly in the cytoplasm of Bel-7402 and SMMC-7721cells when transfected with ITGB4-vector, while a decreased ITGB4 and Slug expression is shown in the MHCC-97H and MHCC-LM3 cells with the silencing of ITGB4. (**E**) Western blot detection of the expression of ITGB4, Slug and the EMT markers E-cadherin, N-cadherin, and vimentin in the Bel-7402 or SMMC-7721 cell lines respectively treated with ITGB4 vector and control vector, and MHCC-97H or MHCC-LM3 cell lines respectively treated with shRNA-ITGB4 and negative control. (**F,G**) Signal pathway of PI3K/AKT and pluripotency factors Sox2 and Nanog were detected using western blot. (**H**) In western blots of BEL-7402 and SMMC-7721 cells overexpressing ITGB4 and treated or untreated with PI3K inhibitor LY294002, a reduction in levels of EMT protein markers and an increase in E-cadherin in cells treated with inhibitor as shown. *P < 0.05, **P < 0.01.

To further evaluate the effect of ITGB4 on HCC tumorigenesis, ITGB4 was ectopically expressed in the Bel-7402 and SMMC-7721 cell lines, which showed moderate ITGB4 overexpression. The results of MTT assay showed that ITGB4 significantly increased cell viability in both cell lines (Fig. 3A), and resulted in an approximately 3-fold increase in the colony forming ability of HCC cells (Fig. 3B). Invasion assays showed that ectopic expression of ITGB4 caused an approximately 2.75-fold increase in the invasive ability of Bel-7402 and SMMC-7721 cells (Fig. 3C). The mechanisms underlying the tumorigenic effects of ITGB4 were further examined by assessing the expression of EMT markers and that of the transcription factor Slug in response to ITGB4 overexpression. Immunofluorescence staining showed that ectopic expression of ITGB4 upregulated Slug in both Bel-7402 and SMMC-7721 cell lines while the expression of Slug and ITGB4 in MHCC-97H and MHCC-LM3 cells transfected with sh-ITGB4 is reduced to that of control cells (Fig. 3D). Furthermore, the results reveal that expression of ITGB4 and Slug are mainly in the cytoplasm of the HCC cells. Western blot analysis showed that ITGB4 overexpression downregulated the epithelial marker E-cadherin and upregulated the mesenchymal markers N-cadherin and vimentin in parallel with the activation of p-AKT and upregulation of Slug, Sox2 and Nanog in Bel-7402 or SMMC-77721 cells, and a contrary results exhibited in the MHCC-97H and MHCC-LM3 cells with the silencing of ITGB4 (Fig. 3E–H), indicating that ITGB4 induces EMT by upregulating Slug and other components of the ITGB4/Slug functional interaction in HCC cells. Moreover, in Bel7402 and SMMC-77721 treated with a P13K inhibitor there is a reduction in the level of EMT proteins and an increase in the levels of E-cadherin indicating that PI3K/AKT pathway mignt be involved in the ITGB4/Slug interaction.

**Figure 4:**
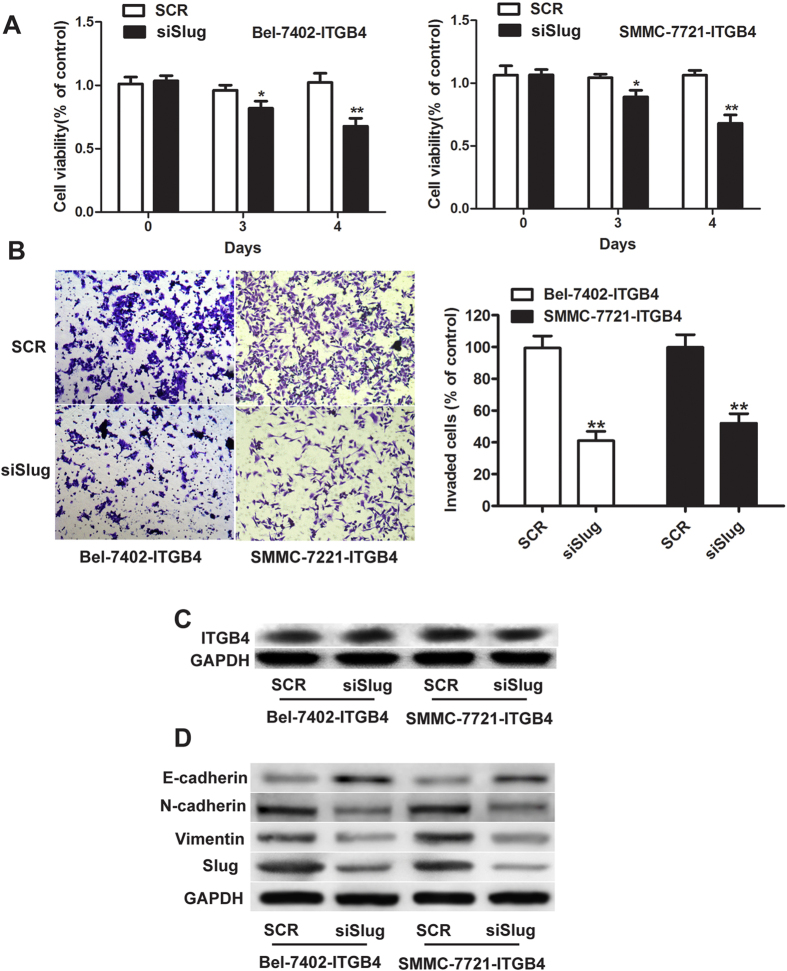
Effects of silencing of Slug on ITGB4 overexpression-induced EMT, cell proliferation and invasion. (**A**) Bel-7402 and SMMC-7721 cells stably overexpressing ITGB4 were transfected with small interfering RNA (siRNA) against Slug, and cell viability was assessed using the MTT assay, and showed significantly lower rates of cell proliferation compared with scramble-siRNA. (**B**) and invasive ability of siSlug-transfected cells was decreased as determined by the Transwell assay and quantified after 24 h compared with scramble-siRNA. **P < 0.01. (**C,D**) The protein expression of ITGB4, Slug and the EMT markers E-cadherin, N-cadherin, and vimentin was determined by western blotting in Bel-7402- ITGB4 and SMMC-7721- ITGB4 cells treated with Slug-siRNA or scramble-siRNA.

To further examine the role of Slug in ITGB4-mediated invasion and EMT, Bel-7402 and SMMC-7721 cells were transfected with siRNA targeting Slug and subjected to cell viability assays using MTT, Transwell invasion and western blot assays. Cell viability was significantly reduced after 4 days in both cell types when Slug was silenced compared with the scrambled control (Fig. 4A, P < 0.01). Slug knockdown in ITGB4-overexpressing cells resulted in an approximately 60–65% decrease in cell invasion (Fig. 4B, P < 0.01), and upregulated E-cadherin and downregulated N-cadherin and vimentin, which has no significant effect on the expression of ITGB4 by WB analysis (Fig. 4C,D). Taken together, these results indicate that the effects of ITGB4 on promoting cell proliferation and invasion and inducing EMT are dependent on the activity of the transcription factor Slug, and AKT/Sox2-Nanog pathway may be involved in it.

### ITGB4 promotes tumor growth and metastasis *in vivo*

**Figure 5:**
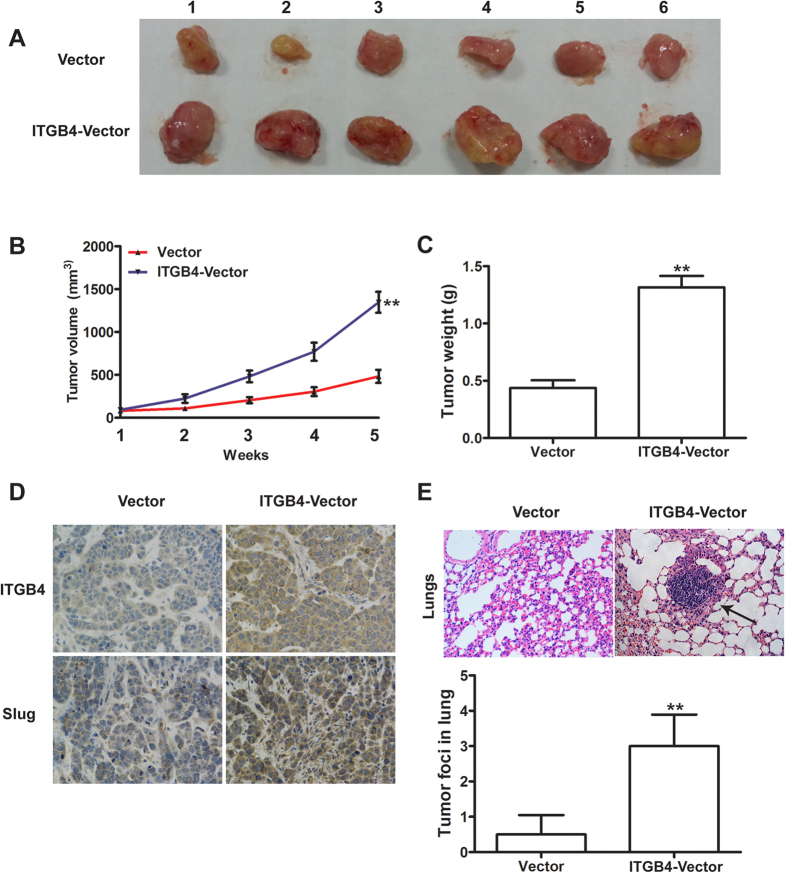
Effects of ITGB4 overexpression on HCC growth and metastasis *in vivo*. (**A**) Representative images of the xenograft tumors formed in nude mice (n = 6 per group) injected with empty vector or ITGB4-transduced cells. (**B,C**) Growth curve and weight of xenograft tumors, exhibiting both significantly greater volume and weight of tumors derived from nude mice injected with ITGB4-transduced cells compared with control after 5 weeks. **P < 0.01. (**D**) Immunohistochemical staining for ITGB4 and Slug in tumor tissues from mice with subcutaneous HCC implantation, demonstrating significantly ITGB4-overexpression and Slug-overexpression in tumor tissues derived from nude mice injected with ITGB4-transduced cells compared with control (original magnification: 400 × ). (**E**) Representative H&E staining of lung metastatic foci from mice injected with vector control or ITGB4-transduced cells, exhibited an approximately 3-fold higher incidence of metastatic foci in mice injected with ITGB4-transduced cells than in those bearing vector control tumors. **P < 0.01.

The effect of ITGB4 was assessed in a xenograft tumor model induced by injection of ITGB4-overexpressing Bel-7402 cells into nude mice. Figure 5A shows representative images of tumors excised from mice injected with vector control or ITGB4 overexpressing cells. Tumor volume and weight were significantly higher in mice injected with ITGB4 overexpressing cells (Fig. 5B,C) (P < 0.01). Immunohistochemical staining showed that ITGB4 and Slug were more strongly expressed in ITGB4-overexpressing tumor tissues than in vector control derived tumors (Fig. 5D), and a relative immunohistochemical panel of all the excised masses showed as supplementary material (see supplementary Fig. S1). Tumor metastasis to the lung was also significantly higher when tumor formation was induced by ITGB4 HCC cells compared with control HCC cells (Fig. 5E). Furthermore, the detection of tumor foci in the lungs of model mice showed an approximately 3-fold higher incidence of metastatic foci in mice injected with ITGB4-overexpressing cells than in those bearing vector control tumors (Fig. 5E). Taken together, these results confirm that ITGB4 exerts tumorigenic and metastatic effects in an *in vivo* model.

## Discussion

The failure of treatment and the mortality of HCC are primarily associated with invasion, metastasis, and recurrence after surgical resection^32^,^33^. The integrin family of receptors plays an important role in the regulation of cellular functions essential to the initiation, progression, and metastasis of solid tumors, which has made these receptors an attractive target for the treatment of cancer^34^. Integrin αVβ5 was reported in osteosarcoma cells which was involved in promotion of the EMT with signal transduction pathway included Raf-1, MEK, ERK, and Elk-1^35^. In metastatic prostate cancer, integrin αVβ3 and CD44 pathways support osteoclastogenesis via a Runx2/Smad 5/receptor activator of NF-κB ligand signaling axis^36^. Similarly, several reports about integrin receptors αVβ3, αVβ5, αVβ1 etc along with CD44 have been shown to play important role in EMT and metastasis^37^,^38^,^39^. Moreover, a5 integrin subunit and b1 integrins were involved in the effect of hepatitis B virus HBx on tumor migration^2^. And integrin aVb3 and CD44 involved in the regulation of EMT through FAK, Src, and Akt in HCV-infected cells^3^. Thus, integrin inhibitors such as peptides and monoclonal antibodies are being investigated in clinical trials for the treatment of solid tumors such as colorectal cancer, renal cell carcinoma, and melanoma, and a specific integrin antagonist has shown promising activity in patients with glioblastoma^40^. Integrins span the lipid bilayer of cells and are involved in several signaling pathways, underscoring the importance of crosstalk between integrins and growth and associated factors in tumor initiation and progression^34^. In the present study, we examined the effect of ITGB4 and its association with the transcription factor Slug on HCC proliferation, invasion, and metastasis *in vitro* and *in vivo* and assessed their potential value as prognostic biomarkers for HCC.

Analysis of ITGB4 expression in HCC showed that it was expressed at higher levels in HCC tissues and cell lines than in adjacent non-tumor tissues and normal hepatic cells. Silencing of ITGB4 in cells expressing high endogenous levels of the receptor inhibited cell proliferation, invasion, and colony formation ability. Alterations in ITGB4 expression have been linked to several malignancies, although its role remains controversial. In breast cancer, ITGB4 overexpression is associated with aggressive behavior and poor prognosis, and ITGB4 was shown to be a prognostic indicator of poor survival and to correlate with nuclear grade and tumor size in breast cancer, cervical cancer, head and neck cancer, and pancreatic cancer^41^,^42^,^43^,^44^. In head and neck cancer, increased ITGB4 expression is associated with lymph node metastasis, distant metastasis, and increased mortality^45^. In pancreatic adenocarcinoma, increased ITGB4 expression is correlated with a number of EMT hallmarks, including solitary cell infiltration, reduced expression of E-cadherin, and increased expression of vimentin^46^. However, the correlation between ITGB4 expression and tumorigenesis and invasion is controversial in many cancers, including colon cancer, ovarian cancer, prostate cancer, and in tumors of the central nervous system^11^. The association of ITGB4 with poor prognosis in many cancers is attributed to the ability of integrin α6β4 to promote malignant behaviours, such as proliferative signaling, the evasion of apoptosis, tissue invasion and metastasis, and the induction of angiogenesis. However, the relation of ITGB4 expression with prognosis in some tumors and not in others remains unclear.

In the present study, we examined the mechanisms underlying the effects of ITGB4 by overexpressing the receptor in HCC cells with moderate endogenous ITGB4 expression. Our results showed that ITGB4 promoted proliferation, invasion, colony formation, and induced EMT in parallel with the upregulation of Slug. The involvement of Slug was confirmed by siRNA-mediated silencing experiments, which showed that Slug knockdown reversed the effect of ITGB4 overexpression, restoring the mesenchymal phenotype and inhibiting cell invasion. Moreover, the confocal immunofluorescent detection of HCC cells showed that the expression of ITGB4 upregulated the level of Slug mainly in the cytoplasm. Although Slug is widely known as a transcription factor that reside in the nucleus, increasing evidence suggests the significance of its cytosolic expression in cancer progression and metastasis^47^,^48^. It is likely that the interaction between two proteins alters its confirmation similar to the previously reported by Luanpitpong S, *et al*. And the stabilization process is consistent with the previous report showing an increase in protein stability by protein-protein interaction and inhibition of protein ubiquitination^49^. In addition, the involvement of PI3K/AKT signal pathway was confirmed by the inhibitor of PI3K in the ITGB4 overexpressing cells. The results showed upregulated the epithelial marker E-cadherin and downregulated the mesenchymal markers N-cadherin and vimentin with inhibition of phosphorylated AKT. Pluripotency factors Sox2 and Nanog were also involved in the ITGB4/Slug interaction as shown by the western blot analysis. These results indicated that ITGB4 may modulate the expression of EMT-inducing transcription factor Slug through the AKT/Sox2-Nanog pathway in HCC.

The effects of the α6β4 integrin on cancer cell migration and invasion have been studied extensively using different approaches, such as the exogenous expression of ITGB4, its siRNA-mediated silencing, or the expression of ITGB4 mutants^50^,^51^,^52^. In addition, α6β4 has been shown to play a role in protein translation by promoting the phosphorylation of the 4E-binding protein (4E-BP) by mammalian target of rapamycin (mTOR), resulting in the disruption of its interaction with the translation initiation factor eIF-4G and the inhibition of protein translation^53^. α6β4 modulates the translation of vascular endothelial growth factor via this mechanism in breast carcinoma cells^54^. Integrin α6β4 has also been suggested to play a role in the regulation of the transcription of genes involved in invasion. The transcription factor nuclear factor of activated T-cells (NFAT) was shown to be a target of integrin α6β4, and the regulation of the transcriptional activity of NFAT by α6β4 plays a role in carcinoma invasion and metastasis^55^. A role for integrins in transcriptional regulation was proposed in a study that identified α3, β1, and β4 integrins as targets of Slug in keratinocytes and showed that the repression of integrin subunit expression by Slug leads to decreased cell adhesion and proliferation^56^. Slug binds to the integrin promoter, repressing its expression and leading to reduced cell adhesion^57^. Slug was also shown to mediate the effect of transforming growth factor-β1 on EMT and integrin α3β1-mediated migration of human squamous cell carcinoma cells^58^. Slug is a repressor of E-cadherin expression and has a known role in the regulation of EMT and cell migration and invasion. These studies linking Slug to the regulation of integrin expression support the present findings suggesting that the tumorigenic and metastatic effects of ITGB4 are mediated by the activation of Slug. Our results were confirmed *in vivo* in a xenograft tumor model induced by injection of ITGB4-overexpressing cells into nude mice, which showed that ITGB4 promoted tumor growth and metastasis to the lungs.

In conclusion, the present study showed that ITGB4 is overexpressed in HCC and plays a role in the regulation of HCC growth and invasiveness. Our *in vitro* and *in vivo* experiments suggest a potential mechanism underlying the tumorigenic effects of ITGB4 involving the transcription factor Slug through AKT/Sox2-Nanog pathway, as a therapeutic target for the treatment of patients with HCC.

## Additional Information

**How to cite this article**: Li, X.-L. *et al*. Integrin β4 promotes cell invasion and epithelial-mesenchymal transition through the modulation of Slug expression in hepatocellular carcinoma. *Sci. Rep.* **7**, 40464; doi: 10.1038/srep40464 (2017).

**Publisher's note:** Springer Nature remains neutral with regard to jurisdictional claims in published maps and institutional affiliations.

## Supplementary Material

Supplementary Figure S1

## Figures and Tables

**Table 1:** Clinicopathological characteristics of patients with HCC and their associations with ITGB4 expression.

Characteristics	Case number (n = 82)	ITGB4 expression (n,%)		P-value
		High (n, %)	Low (n, %)	
Age (years)				0.262
<55	28	16 (57.1)	12 (42.9)	
≥55	54	30 (55.6)	24 (44.4)	
Gender				0.439
Male	69	37 (53.6)	32 (46.4)	
Female	13	5 (38.5)	8 (61.5)	
Locations				0.221
Left	18	10 (55.6)	8 (44.4)	
Right	23	11 (47.8)	12 (52.2)	
Both	41	23 (56.1)	18 (43.9)	
Stage				0.024
I	14	7 (50)	7 (50)	
II/III	68	36 (52.9)	32 (47.1)	
Tumor number				0.060
Single	73	38 (52.1)	35 (47.9)	
Multiple	9	4 (44.4)	5 (55.6)	
HBV				0.083
No	33	17 (51.5)	16 (48.5)	
Yes	49	27 (55.1)	22 (44.9)	
Liver Cirrhosis				0.055
No	7	3 (42.9)	4 (57.1)	
Yes	75	39 (52.0)	36 (48.0)	
Vascular invasion				0.016
No	59	32 (54.2)	27 (45.8)	
Yes	23	13 (56.5)	10 (43.5)
