# Author Correction: Integrin β4 promotes cell invasion and epithelial-mesenchymal transition through the modulation of Slug expression in hepatocellular carcinoma

**DOI:** 10.1038/s41598-023-29191-x

**Published:** 2023-02-06

**Authors:** Xiao-Long Li, Lin Liu, Dan-Dan Li, Ya-Ping He, Le-Hang Guo, Li-Ping Sun, Lin-Na Liu, Hui-Xiong Xu, Xiao-Ping Zhang

**Affiliations:** 1 Department of Medical Ultrasound, Shanghai Tenth People’s Hospital, Ultrasound Research and Educational Institute, Tongji University School of Medicine, Shanghai, 200072 China; 2 Department of Interventional & Vascular Surgery, Tongji University School of Medicine, Shanghai, 200072 China

Correction to: *Scientific Reports* https://doi.org/10.1038/srep40464, published online 13 January 2017

This Article contains an error in Figure 3(C) and (E) and 5(E) where original biological duplicate data results were incorrect.

The correct Figure 3 and 5 and their accompanying legends appear below.

**Figure 3:**
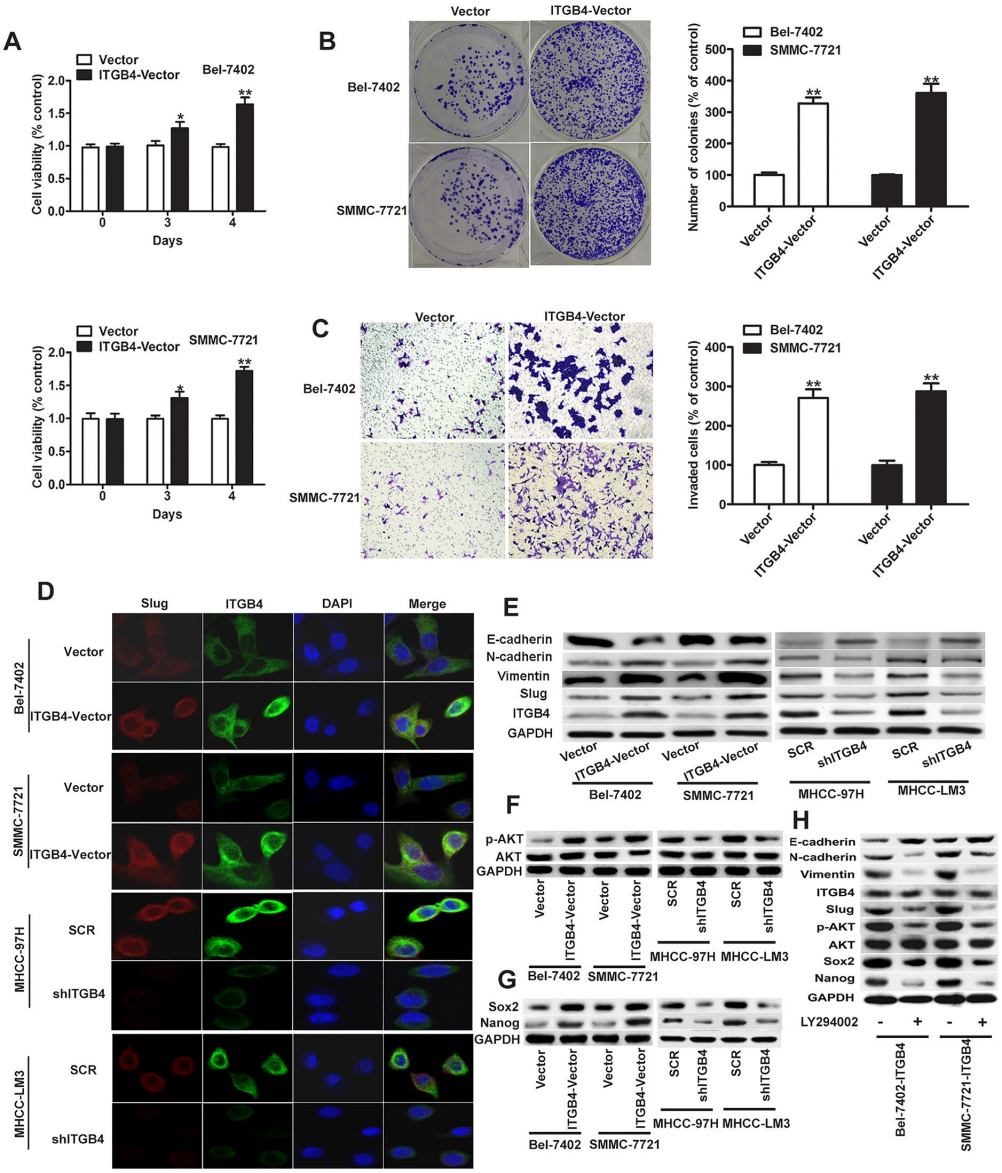
Effects of ITGB4 overexpression on HCC cell proliferation, colony formation, invasion and EMT. (**A**) ITGB4 was ectopically expressed in the Bel-7402 and SMMC-7721 cell lines, and cell viability was assessed using the MTT assay, and showed significantly higher rates of cell proliferation compared with negative control. (**B**) Colony forming ability increased in cells treated with ITGB4 vector, which was determined by crystal violet staining and quantified by counting the number of colonies. (**C**) Compared with negative control, invasive ability of ITGB4-transduced cells was increased determined by the Transwell assay and quantified after 24 h, respectively. (**D**) Representative images of immunofluorescent detection of ITGB4 and Slug expression in ITGB4-overexpressing cells (Bel-7402 and SMMC-7721), sh-ITGB4 cells (MHCC-97H and MHCC-LM3) and matched control cells. Nuclei were counterstained using DAPI. The results demonstrated an increasing expression of ITGB4 and Slug mainly in the cytoplasm of Bel-7402 and SMMC-7721cells when transfected with ITGB4-vector, while a decreased ITGB4 and Slug expression is shown in the MHCC-97H and MHCC-LM3 cells with the silencing of ITGB4. (**E**) Western blot detection of the expression of ITGB4, Slug and the EMT markers E-cadherin, N-cadherin, and vimentin in the Bel-7402 or SMMC-7721 cell lines respectively treated with ITGB4 vector and control vector, and MHCC-97H or MHCC-LM3 cell lines respectively treated with shRNA-ITGB4 and negative control. (**F**,**G**) Signal pathway of PI3K/AKT and pluripotency factors Sox2 and Nanog were detected using western blot. (**H**) In western blots of BEL-7402 and SMMC-7721 cells overexpressing ITGB4 and treated or untreated with PI3K inhibitor LY294002, a reduction in levels of EMT protein markers and an increase in E-cadherin in cells treated with inhibitor as shown. *P < 0.05, **P < 0.01.

**Figure 5:**
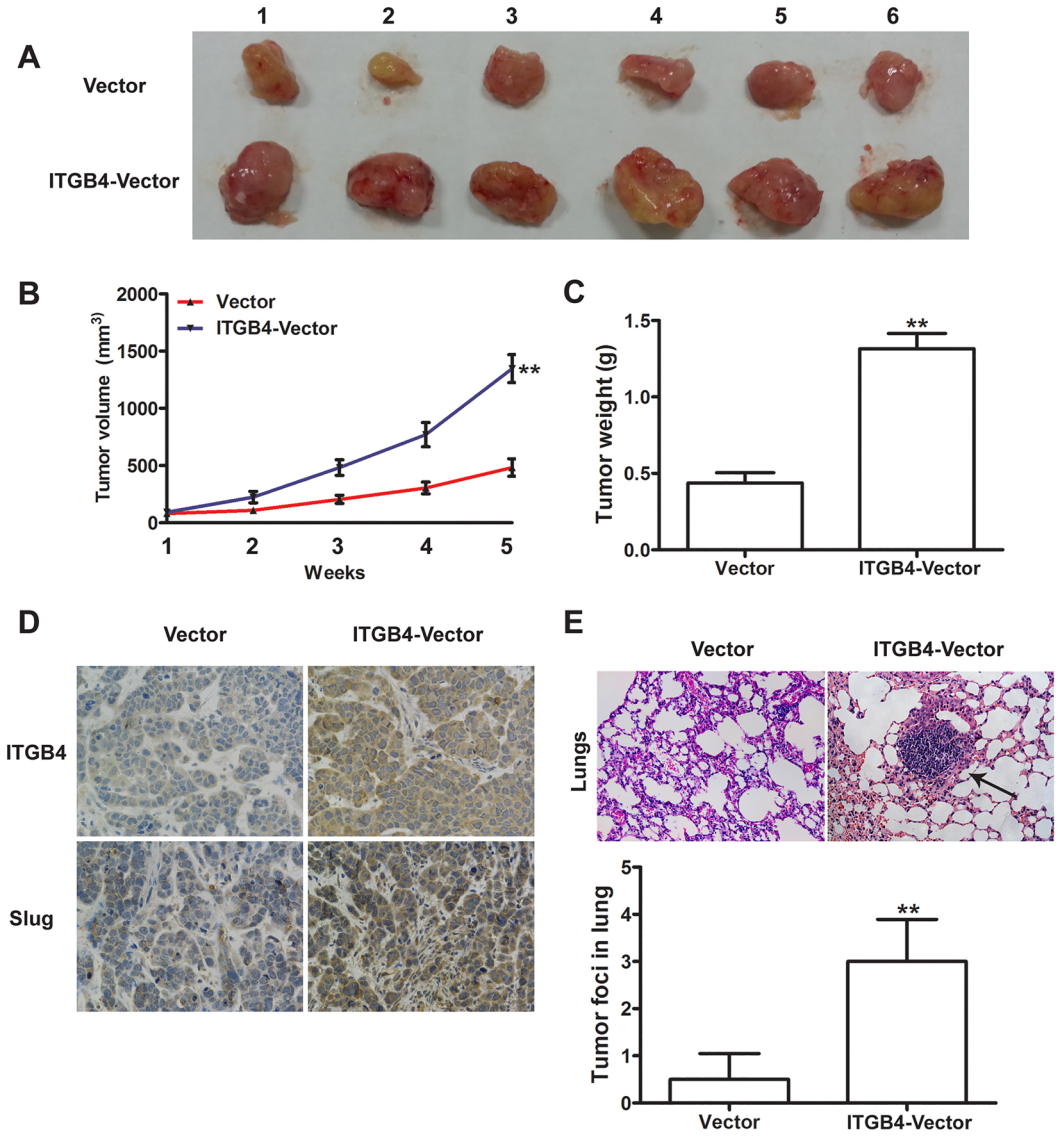
Effects of ITGB4 overexpression on HCC growth and metastasis in vivo. (**A**) Representative images of the xenograft tumors formed in nude mice (n = 6 per group) injected with empty vector or ITGB4-transduced cells. (**B**,**C**) Growth curve and weight of xenograft tumors, exhibiting both significantly greater volume and weight of tumors derived from nude mice injected with ITGB4-transduced cells compared with control after 5 weeks. **P < 0.01. (**D**) Immunohistochemical staining for ITGB4 and Slug in tumor tissues from mice with subcutaneous HCC implantation, demonstrating significantly ITGB4-overexpression and Slug-overexpression in tumor tissues derived from nude mice injected with ITGB4-transduced cells compared with control (original magnification: 400×). (**E**) Representative H&E staining of lung metastatic foci from mice injected with vector control or ITGB4-transduced cells, exhibited an approximately 3-fold higher incidence of metastatic foci in mice injected with ITGB4-transduced cells than in those bearing vector control tumors. **P < 0.01.

This change does not affect the conclusions of the Article.

