# Supplementary material for: Integrin β4 promotes cell invasion and epithelial-mesenchymal transition through the modulation of Slug expression in hepatocellular carcinoma
Source: Sci Rep. 2017 Jan 13;7:40464. doi: 10.1038/srep40464 (PMC5233967; doi:10.1038/srep40464)

Integrin β4 promotes cell invasion and epithelial-mesenchymal transition through the modulation of Slug expression in hepatocellular carcinoma

Xiao-Long Li[1,#], Lin Liu[2,#], Dan-Dan Li[1], Ya-Ping He[1], Le-Hang Guo[1], Li-Ping Sun[1], Lin-Na Liu[1], Hui-Xiong Xu[1,*], Xiao-Ping Zhang[2,*]

[1] Department of Medical Ultrasound, Shanghai Tenth People's Hospital, Ultrasound Research and Educational Institute, Tongji University School of Medicine, Shanghai 200072, China

[2] Department of Interventional & Vascular Surgery, Tongji University School of Medicine, Shanghai 200072, China

# Xiao-Long Li and Lin Liu equally contributed to this work.

*Corresponding author:

Hui-Xiong Xu, Department of Medical Ultrasound, Shanghai Tenth People's Hospital, Ultrasound Research and Educational Institute, Tongji University School of Medicine, Shanghai 200072, China. E-mail: xuhuixiong@126.com. Tel: 86-21-66307539; Fax: 86-21-66307539

Xiao-Ping Zhang, Department of Interventional & Vascular Surgery, Tongji University School of Medicine, Shanghai 200072, China. E-mail: zxpsibs@163.com. Tel: 86-21-64373365; Fax: 86-21-64373365

Figure S1. Immunohistochemical staining for ITGB4 and Slug in the excised tumor tissues shown in panal A , demonstrating significantly ITGB4-overexpression and Slug-overexpression in tumor tissues derived from nude mice injected with ITGB4-transduced cells compared with control (original magnification: 200×).

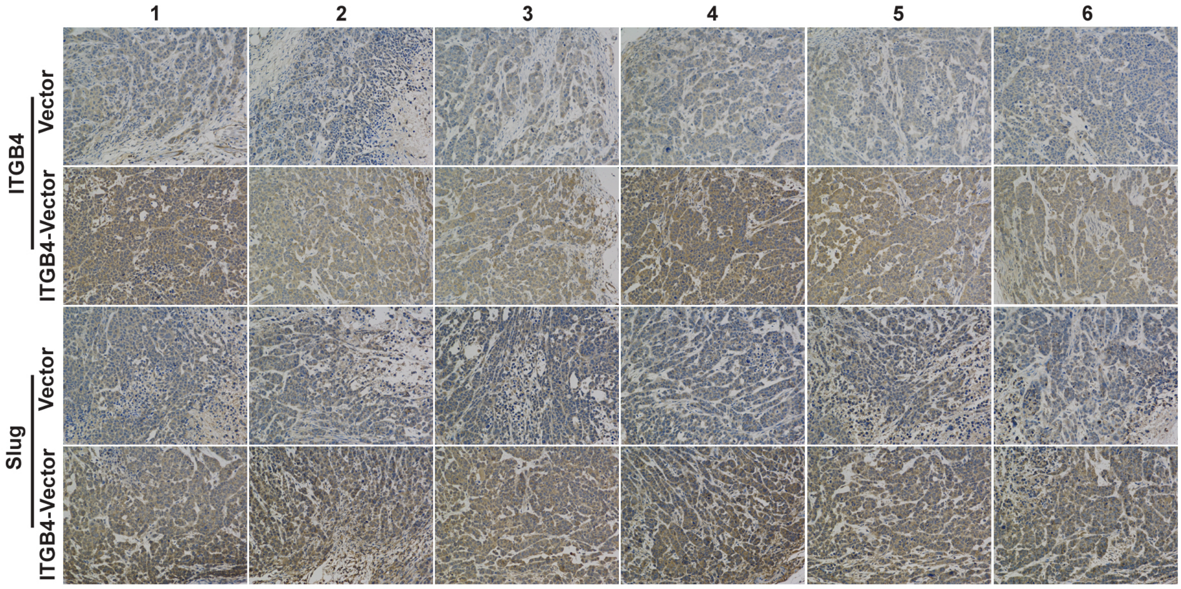

Supplement: Supplementary Figure S1 [file srep40464-s1.pdf]
